# The Electronic Properties and Adsorption Performance of LDH/Graphene, and LDH/g-C_3_N_4_ for the Removal of Pharmaceutical Contaminants: A Molecular Dynamics Simulation

**DOI:** 10.3390/ijms252312730

**Published:** 2024-11-27

**Authors:** Qusai Ibrahim, Salem Gharbia

**Affiliations:** School of Engineering and Design, Atlantic Technological University, Ash Lane, F91 YW50 Sligo, Ireland; qusai.ibrahim@research.atu.ie

**Keywords:** graphene, G-C_3_N_4_, LDH, pharmaceutical contaminants, simulation, adsorption

## Abstract

Water shortages and pharmaceutical pollution are two interconnected crises that pose severe threats to global health, environmental sustainability, and economic stability. Pharmaceutical pollution is widespread and has reached potentially toxic levels in over 258 rivers in 104 countries. So far, more interest has been paid towards efficient water treatment processes in recent years. In this study, we explore the efficacy of layered double hydroxide (LDH) nanocomposites with graphene and graphitic carbon nitride (g-C_3_N_4_) as promising adsorbents of pharmaceutical contaminants. The LDH nanocomposite has been designed and simulated for the first time, consisting of two layers of sodium hydroxide with a layer of graphene and g-C_3_N_4_. We investigated the adsorption performance of LDH, specifically LDH/graphene and LDH/g-C_3_N_4_, for the removal of pharmaceutical contaminants including acetaminophen (AC), caffeine (CAF), and sulfamethoxazole (SMZ). Through comprehensive molecular dynamics simulations using the reactive forcefield (ReaxFF) software, we investigated the adsorption mechanisms, kinetics, and adsorption capacity of pharmaceutical contaminants onto these nanocomposite surfaces. Our findings showed that the combination of LDH/graphene had a higher adsorption capacity for the removal of pharmaceutical contaminants than LDH/g-C_3_N_4_. At 70 Picoseconds (Ps), 124, 129, and 142 molecules of each of the pharmaceutical contaminants AC, CAF and SMZ, respectively, had been adsorbed by LDH/graphene, with a higher exothermic energy equating to −1111, −1015, and −1150 × 10^3^ kJ/mol, respectively. On the other hand, for LDH/g-C_3_N_4_ at 70 Ps, 108, 110, and 120 molecules of AC, CAF and SMZ, respectively, had been adsorbed, with exothermic energy equating to −978, −948, and −1173 × 10^3^ kJ/mol, respectively. Finally, we calculated the electronic properties, including the band gap and density of state of the nanocomposite materials, to check their effect on the adsorption process. In addition, the results showed that the adsorption kinetics followed a pseudo-first-order model, while the adsorption isotherms for AC, CAF and SMZ adhered to the Langmuir model.

## 1. Introduction

Pharmaceutical contaminants are emerging as pollutants of significant concern due to their increasing presence in aquatic environments and drinking water supplies [1]. These contaminants originate from various sources, including human and veterinary drug use, improper disposal of medications, pharmaceutical manufacturing, and agricultural runoff [2]. Once introduced into the environment, pharmaceuticals can persist and accumulate, given their biologically active nature and resistance to conventional wastewater treatment processes [2]. AC, CAF, and SMZ are commonly detected pharmaceutical contaminants in water supplies, posing significant environmental and public health risks [3]. Fram and her colleagues detected pharmaceutical compounds at low concentrations in 2.3% of 1231 samples of groundwater used for public drinking-water supply in California [3]. The detected compounds were AC with a maximum concentration of 1.89 μg/L, CAF with a maximum concentration of 0.29 μg/L, and SMZ with a maximum concentration of 0.17 μg/L [3]. AC, which is widely used as an analgesic and antipyretic, can lead to toxic effects in aquatic organisms, disrupting biological processes even at low concentrations [4]. CAF and SMZ contribute to the growing concern of antibiotic resistance, impacting both aquatic ecosystems and potentially human health through the consumption of contaminated water [5]. The rising presence of these contaminants in water bodies highlights the inadequacies of current water treatment systems, which are often not equipped to effectively remove these complex and diverse pollutants [6]. This growing issue underscores the urgent need for advanced treatment technologies and comprehensive environmental management strategies to mitigate the impact of pharmaceutical contaminants and protect both environmental and public health [7].

Layered double hydroxides (LDHs) have garnered significant attention as promising materials for environmental remediation due to their unique structural features, high anion exchange capacity, and tunable chemical properties [8]. Their structure imparts several advantages to LDHs as adsorbents [8]. First, the high surface area and tunable interlayer spacing of LDHs facilitate the adsorption of a wide range of contaminants, including anionic and cationic species [9]. The ability to easily modify the chemical composition of the metal layers and the type of interlayer anions allows for adsorption properties to be tailored to target specific pollutants [9]. Furthermore, LDHs exhibit high thermal and chemical stability, making them suitable for use in diverse environmental conditions [10]. Their regenerative capacity through simple ion exchange processes ensures the potential for multiple reuse cycles, enhancing their cost-effectiveness [10]. Additionally, the relatively low toxicity and environmental friendliness of LDHs contribute to their appeal as sustainable materials for water and wastewater treatment applications [11]. These combined properties make LDHs highly effective for the removal of pharmaceutical contaminants and other pollutants from aqueous solutions [12].

Furthermore, LDH/graphene demonstrates superior adsorption capabilities due to its higher surface area and stronger π-π interactions, while LDH/g-C_3_N_4_ benefits from hydrogen bonding and electrostatic interactions [13]. When combined with other materials, such as graphene and graphitic carbon nitride (g-C_3_N_4_), LDHs form composites that potentially exhibit their superior adsorption characteristics [14]. Graphene, known for its exceptional surface area and electronic properties, and g-C_3_N_4_, recognized for its chemical stability and photocatalytic activity, can synergistically enhance the adsorption performance of LDHs [15,16]. For instance, Khataee and his colleagues fabricated NiFe layered double hydroxide/reduced graphene oxide through a hydrothermal method for the degradation of moxifloxacin [17]. The results showed a high degradation efficiency of moxifloxacin by using a combination of ultrasound and photocatalysis (sonophotocatalysis) [17]. Along the same lines, Iqbal and his colleagues synthesized MgAl-LDH/graphene protective film to investigate the adsorption behavior of graphene and the effect of graphene on LDH’s physicochemical properties [18]. The results showed that graphene effectively sealed LDH’s micropores, improving its barrier properties and enhancing its corrosion resistance properties [18].

On the other hand, the combination of LDH and g-C_3_N_4_ has improved adsorption capacities, as indicated in previous studies [19,20,21]. For instance, Di and his colleagues investigated the enhancement of the photocatalytic performance of g-C_3_N_4_ using ZnFe-LDH, for the degradation of pharmaceutical pollutants [22]. The results showed that the composite with a low g-C_3_N_4_ content exhibited optimum photocatalytic activity for ibuprofen degradation, while a high g-C_3_N_4_ content was optimal for sulfadiazine degradation due to different dominant reactive species [22]. Moreover, Zhang and his colleagues developed a heterostructure NiCo-LDH/g-C_3_N_4_ for the efficient adsorption and photoreduction of toxic hexavalent chromium ions (Cr (VI) and Congo red (CR) pollutants under visible light irradiation [23]. The NiCo-LDH/g-C_3_N_4_ composites showed fast chromium removal efficiency, high adsorption capacity, and excellent reusability [23].

The use of adsorption for the removal of pharmaceutical contaminants from water presents several challenges and considerations in real-world applications [24]. Technological constraints include the need for efficient adsorbent materials that can selectively target pharmaceutical compounds, while maintaining performance over multiple cycles of use [25,26]. Developing such materials can be limited by cost and the complexity of scaling lab-based solutions to industrial levels [27]. Safety and environmental concerns are also significant, as the disposal or regeneration of adsorbents must ensure that they do not introduce secondary pollutants or harmful byproducts into the environment [28]. LDH/graphene and LDH/g-C_3_N_4_ composites both have environmentally friendly properties [29]. Graphene and g-C_3_N_4_ are generally considered non-toxic and stable, reducing the risk of releasing harmful byproducts during the adsorption process [29]. Moreover, these materials can be regenerated and reused multiple times without significant losses in performance, minimizing waste and reducing environmental impact [29]. While the fabrication of advanced nanomaterials like LDH/graphene and LDH/g-C_3_N_4_ might be more complex than traditional adsorbents, recent advancements in synthesis techniques, such as hydrothermal and solvothermal methods, allow for scalable production of these composites with consistent quality [13]. This addresses the fabrication issue by ensuring that materials can be produced on larger scales without losing their adsorption efficiency or structural integrity [30,31].

In this study, by using molecular dynamic simulation, and with reference to the good adhesion of LDH to graphene and g-C_3_N_4_ in terms of their chemical compatibility [32], a layer of graphene and g-C_3_N_4_ was added to LDH to create a bilayer nanocomposite material, in order to increase its adsorption ability and performance in the elimination of pharmaceutical contaminants including AC, CAF, and SMZ. The adsorption process, van der Waals interactions, adsorption capacity, and potential energy surface (PES) of the composite materials were investigated using the Reactive Forcefield (ReaxFF) software 2022.103, while their electronic properties were calculated using the DFTB code. The adsorption kinetics were evaluated based on the simulation results. The main objective of this project was to develop a complete simulation test for eliminating pharmaceutical contaminants, that can be used in future laboratory experiments.

## 2. Results and Discussion

### 2.1. Adsorption Capacity (qe)

Adsorption capacity is a critical parameter in the field of material science and environmental engineering, reflecting the efficiency and effectiveness of adsorbents in capturing and holding substances on their surfaces [33].

**Figure 1:**
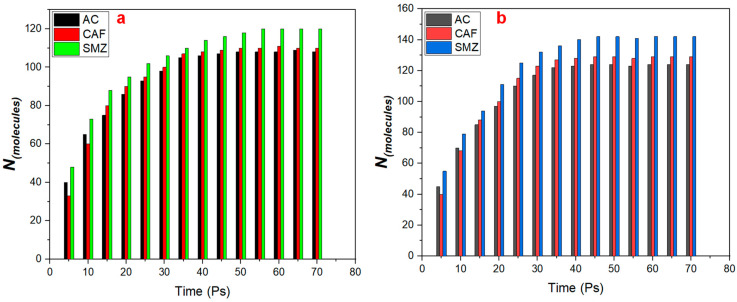
Number of adsorbed AC, CAF, and SMZ molecules on (**a**) LDH/g-C_3_N_4_ and (**b**) LDH/graphene.

As shown in Figure 1, the three pharmaceutical contaminants showed different amounts of adsorbed molecules on LDH/g-C_3_N_4_ and LDH/graphene. SMZ showed the highest amount of adsorbed molecules on both nanocomposite materials, equaling 120 on LDH/g-C_3_N_4_ and 142 on LDH/graphene at t = 70 Ps, which is similar to results reported by a previous study [34]. However, for AC on LDH/g-C_3_N_4_, the number of adsorbed molecules gradually increased for the first 30 Ps until it was equal to 98 molecules, then it increased slowly until it was stable at t = 50 Ps, while on LDH/graphene, the number of adsorbed molecules was equal to 117 at t = 30 Ps, then became stable at t = 40 Ps. For CAF, 100 molecules were adsorbed on LDH/g-C_3_N_4_ at t = 30 Ps, then this increased to 110 molecules at t = 70 Ps, while on LDH/graphene, 123 molecules were adsorbed at t = 30 Ps and this increased to 129 Ps at t = 70 Ps. The number of adsorbed molecules is described in detail in Supplementary Tables S9 and S10.

The adsorption capacity (*qe*) was calculated for the three pharmaceutical pollutants using the following Equation (1) [35,36]:

(1) $$qe=\frac{\left(C_{0}-Ce\right)V}{M}$$

where *q*_e_ is the adsorption capacity, *C*_0_ is the initial concentration (in ppm), *C*_e_ is the concentration at equilibrium, *V* is the volume of solution, and M is the mass of the adsorbent.

**Figure 2:**
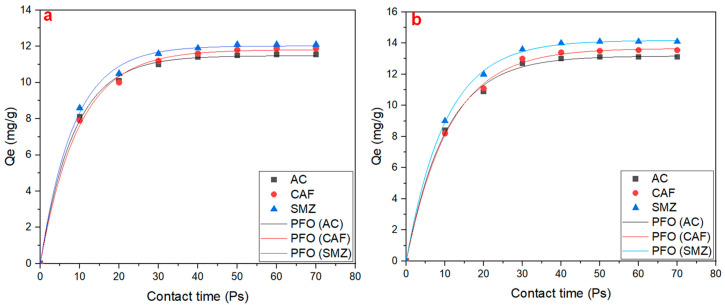
Adsorption capacity of AC, CAF, and SMZ on (**a**) LDH/g-C_3_N_4_ and (**b**) LDH/graphene.

As shown in Figure 2, LDH/graphene had a higher adsorption capacity on the pharmaceutical contaminants after 70 Ps of contact time. For LDH/graphene at t = 20 Ps, the adsorption capacity was equal to 10.9, 11.1, and 12 mg/g, respectively; while for LDH/g-C_3_N_4_, the adsorption capacity was equal to 10.1, 10, and 10.5 mg/g, respectively. However, at t = 30 Ps, there was a significant increase in the adsorption capacity for both LDH/graphene and LDH/g-C_3_N_4_. Finally, at t = 70 Ps, the highest rate of adsorption was reached for both nanocomposite materials, and the relationship remained constant until the end of the simulation time, with a maximum capacity of 13.1, 13.55, and 14.1 for LDH/graphene, while for LDH/g-C_3_N_4_, it was equal to 11.55, 11.88, and 12.1, respectively.

The adsorption kinetics data were adapted into pseudo-first-order (PFO) [37]. The equation for pseudo-first order is shown below:

(2) $$qt=qe(1-e^{-k1t})$$

where *qt* is the amount of adsorbate adsorbed in the time (mg/g), *qe* is the amount of adsorbate adsorbed at equilibrium (mg/g), *k* is the pseudo-first-order rate constant (1/min), and *t* is the time (min). The pseudo-first-order (PFO) was chosen to characterize the adsorption kinetic behaviors. The PFO fit the simulation data with a high R^2^ value of 0.998, 0.998, and 0.997 for AC, CAF, and SMZ on LDH/g-C_3_N_4_, respectively, and 0.998, 0.998, and 0.999 for AC, CAF, and SMZ on LDH/graphene nanocomposite, respectively. Additionally, the calculated qe values from the PFO model were closer to the simulation qe values for both LDH/g-C_3_N_4_ and LDH/graphene (Table 1 and Table 2).

**Figure 3:**
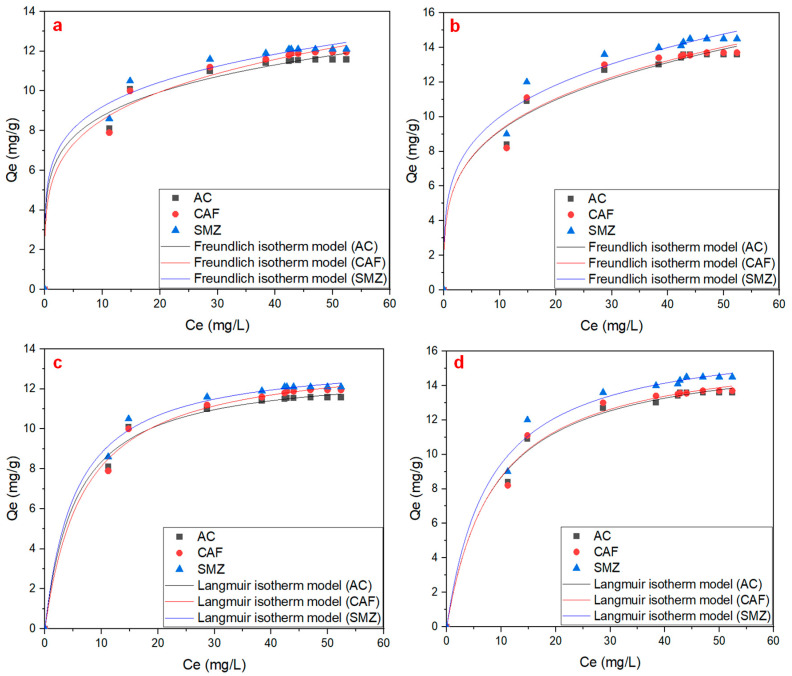
Freundlich adsorption isotherm on (**a**) LDH/g-C_3_N_4_ and (**b**) LDH/graphene. Langmuir adsorption isotherm on (**c**) LDH/g-C_3_N_4_ and (**d**) LDH/graphene.

As shown in Figure 3, the Freundlich and Langmuir isotherm model has been used to describe the adsorption isotherm of AC, CAF, and SMZ on LDH/g-C_3_N_4_ and LDH/graphene. The Freundlich isotherm is an empirical model that assumes that adsorption occurs on a heterogeneous surface with sites of varying affinities [38]. It is expressed by Equation (3) [39]:

(3) $$qe=KF{Ce}^{1/n}$$

*qe*: Amount of adsorbate adsorbed per unit mass of adsorbent at equilibrium (mg/g).

*KF*: Freundlich constant indicative of adsorption capacity ((mg/g)(L/mg)^(1/*n*)^).

1/*n*: Heterogeneity factor indicating adsorption intensity.

Meanwhile, the Langmuir isotherm is a widely used mathematical model that describes the adsorption of molecules onto a solid surface [40]. It is expressed by Equation (4) [40]:

(4) $$qe=\frac{q_{max}bCe}{1+bCe}$$

where *qe* is the amount of adsorbate adsorbed per unit mass of adsorbent at equilibrium (mg/g); *q_max_* is the maximum adsorption capacity, representing the total number of adsorption sites (mg/g); *b* is the Langmuir constant related to the affinity of the binding sites (L/mg); and *C_e_* is the equilibrium concentration of the adsorbate in the solution (mg/L).

The Freundlich isotherm fits the simulation data with a good linear correlation, with a coefficient R^2^ value of 0.866, 0.896, and 0.876 for AC, CAF, and SMZ on LDH/g-C_3_N_4_, respectively, and 0.905, 0.870, and 0.875 for AC, CAF, and SMZ on LDH/graphene nanocomposite, respectively. Meanwhile, the Langmuir isotherm fits the simulation data with a higher linear correlation, with a coefficient R^2^ value of 0.993, 0.993, and 0.994 for AC, CAF, and SMZ on LDH/g-C_3_N_4_, respectively, and 0.992, 0.988, and 0.989 for AC, CAF, and SMZ on LDH/graphene nanocomposite, respectively. The results indicate that the adsorption process of LDH/g-C_3_N_4_ and LDH/graphene for AC, CAF, and SMZ was based on the Langmuir adsorption isotherm model.

Table 3 and Table 4 shows the isotherm parameters of AC, CAF, and SMZ on LDH/g-C_3_N_4_ and LDH/graphene, respectively.

Evaluating the nanocomposite materials presented in this paper against those from other research articles is difficult due to the use of different materials and varying conditions in each study. However, the findings regarding the adsorption capacity, isotherms, and kinetic models were compared with similar research documented in the literature, as detailed in Table 5.

### 2.2. Potential Energy Surface (PES)

PES in the adsorption process is a theoretical representation that describes the variation of the potential energy of a system as a function of the positions of the atoms or molecules involved [47]. The PES illustrates how the energy of an adsorbate molecule changes as it approaches, interacts with, and adheres to the surface of an adsorbent [48]. Mathematically, the PES might be represented as a function of the distance between the adsorbate and the surface and the orientation of the adsorbate [49]:

(5) E=E(d,θ,∅)

where *d* is the distance between the adsorbate and the surface, and *θ* and ∅ are angles describing the orientation of the adsorbate relative to the surface. In this study, we calculated the MD-PES for both LDH/g-C_3_N_4_ and LDH/graphene. Due to the attraction between AC, CAF, and SMZ and the surface of the nanocomposite materials, the results showed a negative potential energy for the molecules equal to −948, −978, and −1173 MJ/mol for AC, CAF, and SMZ, respectively, on LDH/g-C_3_N_4_ at t = 40 Ps. The reason for the negative potential energy can be expressed according to Coulomb’s Law [50]:

(6) $$F=\frac{k\cdot q1\cdot q2}{r^{2}}$$

where *k* is a constant, *q*1 and *q*2 are the quantities of each charge, and the scalar *r* is the distance between the charges.

The $-\frac{k\cdot q1\cdot q2}{r^{2}}$ term in Coulomb’s Law contributes a negative value when *q*1 and *q*2 have opposite signs, indicating an attractive electrostatic force [51].

**Figure 4:**
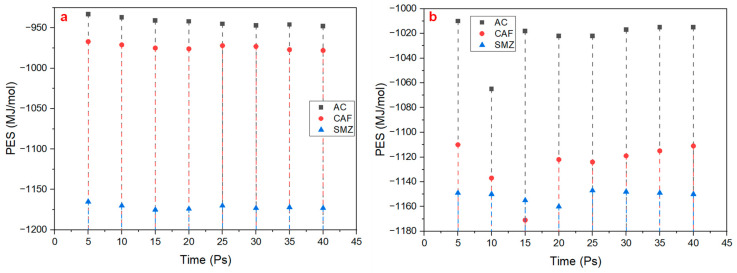
PES during the adsorption process of AC, CAF, and SMZ on (**a**) LDH/g-C_3_N_4_ and (**b**) LDH/graphene.

However, as shown in Figure 4, for LDH/graphene, the potential energy of the molecules was equal to −1015, −1111, and −1150 for AC, CAF, and SMZ at t = 40 Ps, respectively. As the adsorption capacity was higher on LDH/graphene, as we indicated in Section 2.1, the breaking of the molecular bonds and the forming of new bonds and electrons was higher on LDH/graphene, resulting in a lower potential energy and more heat being released (exothermic reaction). Finally, we noticed that the MD-potential energy of AC, CAF and SMZ slightly decreased during the MD-simulation time for LDH/g-C_3_N_4_. The decrease in the potential energy is due to the significant contribution of electrostatic energy [52]. When the atoms’ charge is moving close to the nanocomposite surface, the potential energy decreases [53]. However, on LDH/graphene, the potential energy of both AC and CAF decreased during the first 10 Ps, then it increased until the end of the MD-simulation time, while for SMZ, slightly decreased during the first 20 Ps, then it increased until the end of the MD-simulation time. The increase in the potential energy was due to the hydrogen-bonding sites that formed between the pharmaceutical contaminants and the adsorbent surface [54].

### 2.3. Interaction Energy (Van der Waals Forces)

The interaction energy plays a crucial role in the adsorption process, particularly in physical adsorption (physisorption), where the adsorbate adheres to the surface of the adsorbent without forming strong chemical bonds [55]. Van der Waals forces, which are a specific type of interaction energy, are relatively weak compared to chemical bonds [56]. This allows for the adsorbate to be easily adsorbed and desorbed from the adsorbent surface, making the process reversible [56]. This is important in applications like gas storage and separation, where reversibility is desired [56].

The van der Waals Equation of state is given by [57]:

(7) $$\left(P+\frac{an2}{V2}\right)\left(V-nb\right)=nRT$$

where *P* is the pressure, *R* is the universal gas constant, *T* is the absolute temperature, *V* is the molar volume, and (*a*, *b*) are gas constants.

The Lennard-Jones potential is often used to describe the van der Waals interaction between neutral atoms or molecules. The Equation (8) [57] is given by:

(8) $$E_{LJ}\left(r\right)=4\in [{\left(\frac{\sigma }{r}\right)}^{12}-{\left(\frac{\sigma }{r}\right)}^{6}]$$

where *r* is the distance between the particles, ∈ is the depth of the potential well (related to the strength of the interaction), and *σ* is the finite distance at which the inter-particle potential is zero.

**Figure 5:**
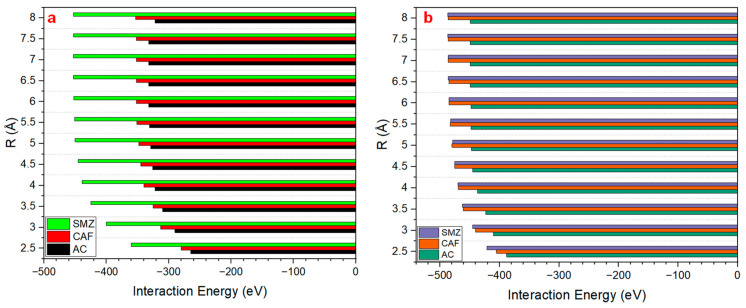
The interaction energy as a function of separation distance on (**a**) LDH/g-C_3_N_4_ and (**b**) LDH/graphene.

In this paper, as shown in Figure 5, we calculated the interaction energy of AC, CAF, and SMZ on LDH/g-C_3_N_4_ and LDH/graphene. We focused on analyzing the change in interaction energy concerning the distance between AC, CAF, and SMZ molecules and the nanocomposite layers. Our results showed that the optimal distance between the three pharmaceutical contaminants and the nanocomposite layers ranged between 2.5 and 3 Å with an interaction energy ranging between −265 and −400 eV for LDH/g-C_3_N_4_, and −388 and −445 for LDH/graphene. However, at *R* = 4 Å, the interaction energy continued to decrease, and it reached a value ranging between −322 and −439 eV for LDH/g-C_3_N_4_, and −437 and −470 eV for LDH/graphene. Finally, for LDH/g-C_3_N_4_, the interaction energy began toward the equilibrium at *R* = 5 Å; meanwhile, for LDH/graphene, the interaction energy began toward equilibrium at *R* = 5 Å for AC, while for CAF and SMZ the interaction energy continued to decrease, and it began toward the equilibrium at R= 6.5 Å. This can be attributed to the hydrogen-bonding sites that formed between CAF and SMZ and the adsorbent surface, as described in Section 2.2.

### 2.4. Adsorption Mechanism of AC, CAF, and SMZ

**Figure 6:**
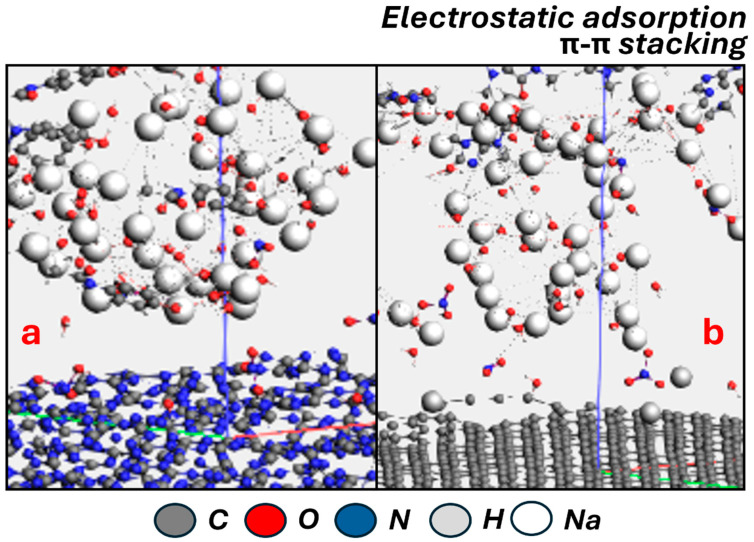
Illustration of adsorption mechanism of AC molecules on (**a**) LDH/g-C_3_N_4_ and (**b**) LDH/graphene.

AC, CAF, and SMZ are adsorbed on LDH/g-C_3_N_4_ and LDH/graphene via a heterogeneous multilayer process, involving p-p stacking interactions between porous activated carbon and the contaminants, which is similar to the results indicated in a previous study [58]. As determined in a previous study [59], it is well known that p-p stacking interactions can explain the adsorption mechanism of aromatic substances on a layered activated carbon surface. Therefore, the molecular structure of the three contaminants enables p-p stacking interactions between the benzene ring (p-electron acceptor) and the p-rich activated carbon adsorbent as indicated in a previous study [60]. However, AC, CAF, and SMZ contain different charged or electron-rich groups, as they act as amphoteric molecules [59]. In addition, electrostatic interactions have been established between the various functional groups of AC, CAF, and SMZ and the corresponding surface structure of porous activated carbon, as we have shown in the previous Section 2.3. Figure 6 and Supplementary Figure S1 show the adsorption mechanism of AC molecules on LDH/g-C_3_N_4_ and LDH/graphene.

### 2.5. Electronic Properties of LDH, g-C_3_N_4_, and Graphene

The electronic properties, including the band gap and DOS, of LDH/graphene and LDH/g-C_3_N_4_ nanocomposite materials have been calculated using the DFTB code based on first-principles density functional theory. For electronic properties, the exchange and correlation interactions were modeled using the Generalized Gradient Approximation functional (GGA) and the Perdew-Burke-Ernzerhof (PBE) functional. The cut-off kinetic energy of the electron wave function will be 489.80 eV, with a good-quality k-point sampling set of 4 × 4 × 7. The PDivision of the reciprocal unit cell based on the Monkhorst-Pack scheme was converged.

**Figure 7:**
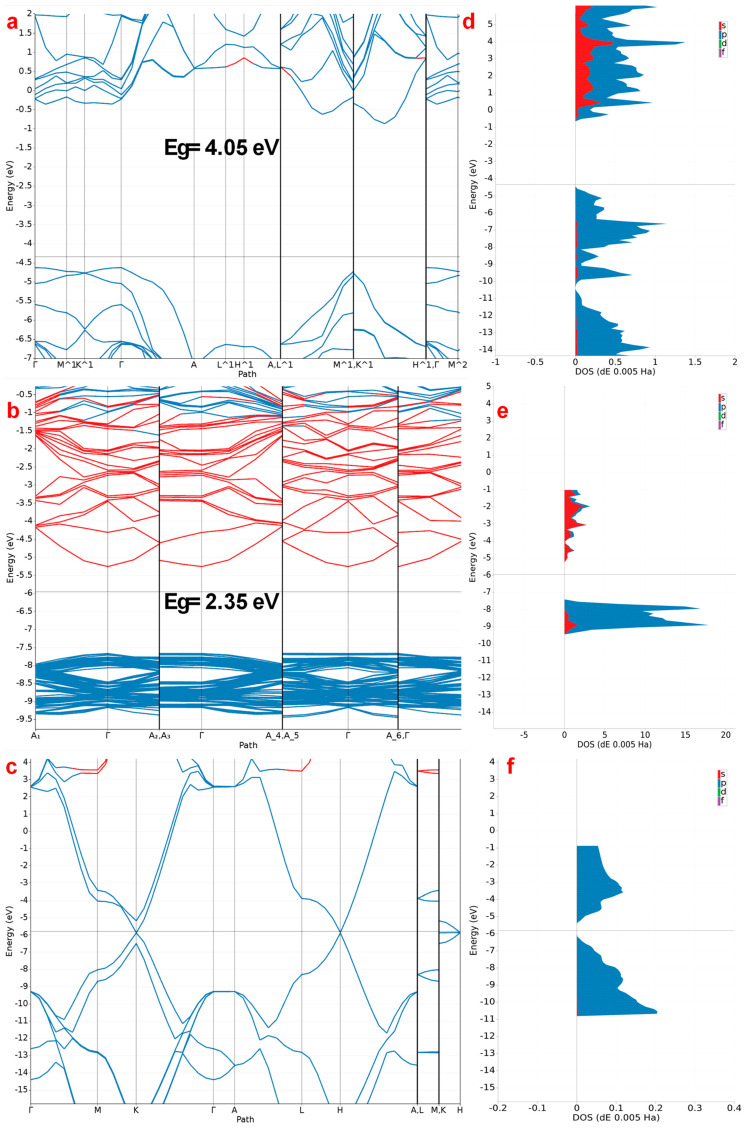
Calculated band structure of (**a**) g-C_3_N_4_, (**b**) LDH, and (**c**) graphene. Calculated DOS of (**d**) g-C_3_N_4_, (**e**) LDH, and (**f**) graphene.

In adsorption techniques, the band gap influences the adsorption capacity and the efficiency of charge transfer processes [61]. Materials with a narrow band gap, such as semiconductors, often facilitate the transfer of electrons between the adsorbate and the adsorbent, enhancing chemisorption through the formation of strong chemical bonds [62]. As shown in Figure 7, the calculated band gaps for g-C_3_N_4_, LDH, and graphene were equal to 4.05 eV, 2.35 eV, and 0.78 eV, respectively, which are similar to the results indicated by previous studies [63,64,65]. The combination of two nanocomposites, one with a wide band gap and one with a narrow band gap, can result in a composite material with an overall reduced band gap [66]. The reduced band gap can improve the material’s ability to absorb a broader spectrum of light and can enhance its photocatalytic and electronic properties, making it more efficient for applications such as photocatalysis, photovoltaic devices, and advanced adsorption techniques [67,68,69].

However, in order to determine the number of electrons in the conduction band and valence band, we calculated the DOS for the three nanocomposite materials. For g-C_3_N_4_, the conduction band is mainly constituted of N-p levels, and the valence band is constituted mainly of N-p and C-s states. For LDH, the conduction band is mainly constituted of Na-p levels, and the valence band is constituted mainly of Na-p and O-s states. Finally, for graphene, the conduction and valence bands are mainly constituted of C-p levels.

## 3. Materials and Methods

### 3.1. Molecular Structure for LDH/g-C_3_N_4_ and LDH/Graphene

**Figure 8:**
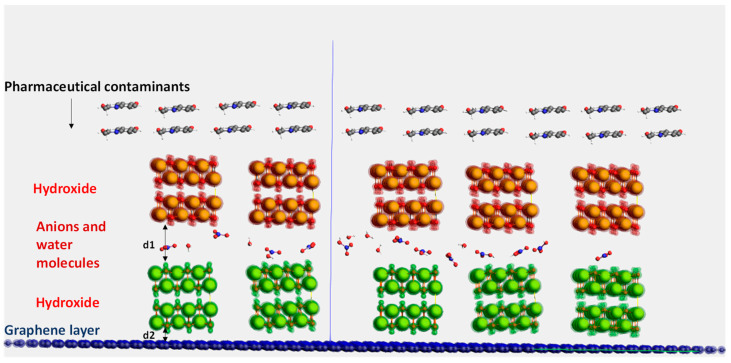
Illustration of the molecular structure of the new nanocomposite material. Carbon atoms in grey, oxygen atoms in red, nitrogen atoms in blue, hydrogen atoms in white, and sodium atoms in yellow. The upper hydroxide layer is highlighted in red, lower hydroxide layer is highlighted in green, and graphene layer is highlighted in blue. (d1 = 14.1 Å), (d2 = 2.7 Å).

The molecular structure for each nanocomposite was simulated based on their crystallographic lattice parameters, as indicated in Supplementary Table S1. For g-C_3_N_4_, a hexagonal crystal system with a space group of P6m_2_ and a cell volume of 87.3 Å^3^ was simulated [70,71], while for graphene, it had a space group of P6_3_/mmc and a cell volume of 1130.6 Å^3^ [72]. For LDH, the molecular structure was designed using the ReaxFF software [73]. A double layer of sodium hydroxide with HNO_3_^−^ anions and H_2_O molecules was optimized using the geometry optimization task in the DFTB Modules in the Amsterdam Modeling Suite (Section 2.3) [74]. The distance between the two hydroxide layers was equal to d1 = 14.1 Å, and the distance between the lower hydroxide layer and the graphene layer was equal to d2 = 2.7 Å, as shown in Figure 8.

Supplementary Table S1 shows the lattice constants for g-C_3_N_4_, graphene, and LDH. However, for the pharmaceutical contaminants, the molecular structure were created using the Amsterdam Modeling Suite software library 2022.103 (Software for Chemistry & Materials, Amsterdam, Netherlands) [72]. For AC (C_8_H_9_NO_2_), the material adopts a monoclinic form with a space group of P2_1_/a and has a density of 1293 g/cm^3^. For CAF, it is described as an orthorhombic form with a space group of P2.2.2. and a density of 1.23 g/cm^3^. Finally, for SMZ, the material presents a monoclinic form with a space group of P2_1_/c and has a density of 1.3915 g/cm^3^. Using the Builder task, the molecules were generated in the simulation box within 2 cycles, with a distance of 2.5 Å and a 50-maximum number of optimization loops. Supplementary Figure S2 shows the initial design of the new nanocomposite materials.

### 3.2. Molecular Dynamics Simulation Using the ReaxFF Software

ReaxFF offers several advantages for studying physisorption, particularly due to its ability to model complex interactions at the atomic level. Its reactive force field framework allows for the exploration of non-covalent interactions, such as van der Waals forces and π-π stacking, which are essential in understanding the adsorption behavior of molecules on various surfaces. ReaxFF can effectively capture the energy landscape associated with physisorption, enabling detailed assessments of adsorption capacities and the stability of adsorbed species. Furthermore, the flexibility of ReaxFF in simulating various environmental conditions makes it an ideal tool for investigating the dynamics of adsorption processes, providing insights that are crucial for the design and optimization of materials for applications such as drug delivery and environmental remediation. In our study, a simulation box consisting of LDH/graphene and LDH/g-C_3_N_4_ nanocomposites with bilayer membranes was designed with a fixed number of 2941 and 3173 atoms, respectively. The membrane surface area was designed with a dimension of 60 × 60 Å^2^, and with a simulation box size of 70 × 70 × 75 Å^3^. For LDH/g-C_3_N_4_ and LDH/graphene, the concentration of LDH was 35.2% from the whole system (LDH/g-C_3_N_4_), while it was 38.8% from the whole system (LDH/graphene). The NHC thermostat simulation method with MTK barostat [75] was applied with a density of 0.255 Kg/L. A force field containing all the elements available in the precursor—(C/H/O/N/Na) for the LDH/g-C_3_N_4_, and (C/H/Na/O) for the LDH/graphene nanocomposite membrane—were selected from the software library [72]. The simulation box’s specific temperature was chosen as 273.15 K with a damping constant equal to 500 fs. An external temperature of 500 K was applied to all the nanocomposite materials in the simulation box during the process. The total simulation time was 500 Ps, which included both the system optimization and convergence phases. The system reached convergence after approximately 400 Ps, ensuring that the atomic positions and energy of the system had stabilized. After this convergence, we focused on studying the adsorption behavior of the organic molecules, conducting simulations for an additional 100 Ps to observe and analyze the adsorption dynamics on the designed nanocomposite bilayer membranes. This approach allowed us to capture a steady-state condition and provide reliable insights into the adsorption process. The results were validated after reviewing the simulation file with a total error equal to 20 bytes from the whole simulation capacity.

**Figure 9:**
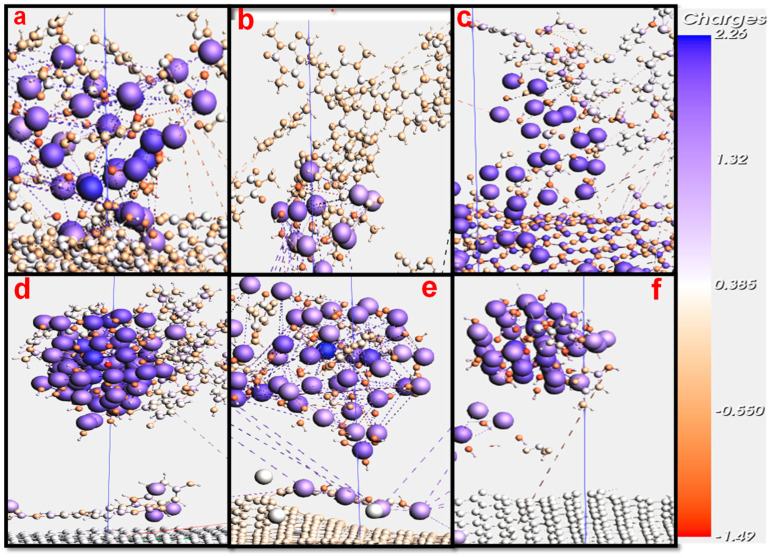
Atomic charges of (**a**) AC, (**b**) CAF, and (**c**) SMZ on LDH/g-C_3_N_4_, and (**d**) AC, (**e**) CAF, and (**f**) SMZ on LDH/graphene, during the adsorption process at t = 10 Ps.

Figure 9 shows an example of the initial configuration of the molecular structure of the new nanocomposite material consisting of 20 AC molecules on the LDH/graphene nanocomposite. We want to mention that the initial configuration for the other pollutants (CAF and SMZ) is the same as for the AC molecules.

### 3.3. Geometry Optimization and Simulation of the Electronic Properties Using DFTB Code

The DFTB code was used to calculate the electronic properties, including band structure and density of state (DOS), of the nanocomposite materials with the same number of atoms and unit cells, as included in Section 2.2. The DFTB code was used because it gives more accurate results, by starting with the pre-optimizer key, and then using the optimum model based on the nanocomposite structure [76,77]. A DFTB3 model [78] with a bulk periodicity with a good-quality K-space has been used. The Fermi temperature was set to 300 K. However, after constructing a crystallographic structure for the nanocomposite materials based on their fundamental lattice characteristics, the geometry optimization task was the first step in the simulation process. With a high number of iterations, totaling 4143 for LDH/graphene and 4500 for LDH/g-C_3_N_4_, the DFTB simulation code was used to complete the process. A Quasi-Newton method was used with a medium-quality process, within an energy range of 2.0 e–5 eV/atom, with a maximum displacement of 0.05 Å. This method was used to minimize the energy used, by arranging the atoms in a certain structure using the lowest possible amount of energy. The geometry optimization parameters for LDH/g-C3N4 and LDH/graphene are presented in Supplementary Tables S2 and S3.

### 3.4. Vibrational Analysis

Infrared (IR) intensity, in the context of molecular spectroscopy, refers to the strength or amplitude of the absorption of infrared radiation by a molecule [79]. The IR intensity provides critical information about molecular vibrations, the nature of chemical bonds, and the molecular structure [79]. It is a key parameter in infrared spectroscopy which is widely used for qualitative and quantitative analysis in chemistry, biology, and materials science [79]. In this section, we will perform a vibrational analysis, and calculate the IR intensity for the optimized structures of LDH, LDH/g-C3N4, and LDH/graphene using the DFTB code. Supplementary Figure S3 shows the calculated vibrational frequencies and IR intensity for LDH, LDH/g-C3N4, and LDH/graphene.

The results showed strong and moderate IR intensities for each nanocomposite material due to the vibrational mode of the nanostructure within the composite. LDH has a total of 318 vibrational modes, while LDH/g-C3N4 and LDH/graphene each have a total of 360 and 414 vibrational modes, respectively. However, the strongest IR peak for LDH, LDH/g-C3N4, and LDH/graphene are located at 608, 2250, and 3700 cm^−1^, respectively, which are similar to the results indicated in previous studies [80,81]. The wave numbers and assignment of bands in the IR spectra of LDH, LDH/g-C3N4, and LDH/graphene are presented in Supplementary Tables S4–S6.

### 3.5. Atomic Charges for LDH/Graphene, and LDH/g-C_3_N_4_ with AC, CAF, and SMZ During the Adsorption Process

Atomic charges play a crucial role in determining the nature and strength of adsorption processes [82]. By understanding and manipulating these charges, it is possible to optimize adsorption for a wide range of industrial and scientific applications [83]. Figure 2 shows the atomic charges of AC, CAF, and SMZ on LDH/g-C_3_N_4_, and LDH/graphene after 10 Ps of simulation time.

As shown in Figure 9, the atomic charges ranged between −0.550e and 0.385e for the AC molecules on both the LDH/g-C3N4 and LDH/graphene nanocomposite materials. However, for CAF, the atomic charges ranged between −0.3e and 0.4e on LDH/g-C3N4, and between −0.9 and −0.5 on LDH/graphene. For SMZ, the atomic charges ranged between −0.550e and 1.32e on LDH/g-C3N4, and between −1.49e and 0.385e on LDH/graphene. However, for AC, CAF, and SMZ at t = 0.5 Ps, the atomic charges for all atoms ranged between 0 and 0.350e. The increments in the atomic charges of the three pharmaceutical contaminants is due to the highly effective nuclear charges which cause a greater attraction to the electrons, resulting in a smaller atomic radius [84], and an increment in adsorption capacity, as has been indicated in a similar study [84]. The atomic charges of AC, CAF, and SMZ on LDH/g-C3N4 and LDH/graphene are described in detail in Supplementary Tables S7 and S8.

## 4. Conclusions

In this study, we investigated the adsorption behavior of LDH consisting of two layers of sodium hydroxide with a layer of graphene and a layer of g-C_3_N_4_, in order to understand the effect of the two layers on the adsorption behavior of LDH. The two nanocomposite materials and the pharmaceutical contaminants AC, CAF, and SMZ have been simulated using the ReaxFF software. Our results found that LDH/graphene nanocomposite had a higher adsorption capacity for the removal of pharmaceutical contaminants than LDH/g-C_3_N_4_ during the total simulation time. During our investigations for the elimination of the pharmaceutical contaminants, we found that SMZ was the highest-adsorbed pharmaceutical contaminant. During the adsorption process, we noticed that the MD-potential energy of AC, CAF and SMZ slightly decreased during the MD-simulation time for LDH/g-C_3_N_4_, while for LDH/graphene, the potential energy of both AC and CAF decreased during the first 10 Ps, then it increased until the end of the MD-simulation time. For SMZ, it slightly decreased in the first 20 Ps, then it increased until the end of MD-simulation time due to the hydrogen-bonding sites that formed between the pharmaceutical contaminants and the adsorbent surface. In addition, our calculations for the electronic properties of the three nanocomposite materials showed that g-C_3_N_4_ and LDH have a wide band gap, while it is narrow for graphene. The combination of these nanocomposites can result in a nanocomposite with an overall reduced band gap for more efficient advanced adsorption techniques. Finally, our results followed a pseudo-first-order model for the adsorption kinetics, while the adsorption isotherms for AC, CAF and SMZ adhered to the Langmuir model. Our study is a strong reference for future research that can explore hybrid nanocomposites, novel synthesis methods, and the integration of machine learning to predict adsorption performance under varying real-world conditions. Additionally, scaling up these findings for industrial water treatment applications will help to bridge the gap between MD-simulations and practical deployment, contributing to more sustainable and efficient water purification technologies. While this study primarily focuses on the adsorption performance of LDH/graphene and LDH/g-C_3_N_4_-based supports, it is essential to acknowledge the importance of the desorption process for a material’s reusability. Considering the high cost of graphene and its derivatives, effective desorption is critical to ensure economic viability and sustainable application in real-world scenarios. Future studies should explore optimized desorption techniques to enhance the reusability and cost-effectiveness of the developed support material.

## Figures and Tables

**Table 1:** Kinetic data-fitting parameters of AC, CAF, and SMZ on LDH/g-C_3_N_4_.

Adsorbent		Pseudo-First-Order		
	q_e_ (mg/g) (Sim)	q_e_ (mg/g) (cal)	k_1_ Ps^−1^	R^2^
AC	11.55	11.48 ± 0.07511	0.024	0.998
CAF	11.88	11.808 ± 0.0949	0.034	0.998
SMZ	12.1	12.024 ± 0.1001	0.042	0.997

**Table 2:** Kinetic data-fitting parameters of AC, CAF, and SMZ on LDH/graphene.

Adsorbent		Pseudo-First-Order		
	q_e_ (mg/g) (Sim)	q_e_ (mg/g) (cal)	k_1_ Ps^−1^	R^2^
AC	13.1	13.17 ± 0.11044	0.044	0.998
CAF	13.55	13.66 ± 0.09701	0.0315	0.998
SMZ	14.1	14.18 ± 0.06512	0.0156	0.999

**Table 3:** Isotherm parameters of the AC, CAF, and SMZ on LDH/g-C_3_N_4_.

Adsorbent	Freundlich			Langmuir	
	K_F_	N	R^2^	Q_e_ (cal)	R^2^
AC	5.69	0.185	0.866	12.99	0.993
CAF	5.15	0.219	0.896	13.70	0.993
SMZ	6.05	0.182	0.876	13.56	0.994

**Table 4:** Isotherm parameters of the AC, CAF, and SMZ on LDH/graphene.

Adsorbent	Freundlich			Langmuir	
	K_F_	N	R^2^	Q_e_ (cal)	R^2^
AC	5.06	0.258	0.905	16.11	0.992
CAF	5.06	0.260	0.870	16.30	0.988
SMZ	5.71	0.242	0.875	16.92	0.989

**Table 5:** Comparison of the results of this paper with the available literature.

Pharmaceutical Contaminants	Simulation Method/Experimental	Kinetic Model	Isotherm Model	Nanocomposite Material	Adsorption Capacity (*qe*) (mg/g)/(mmol/g)	References
AC, CAF, and SMZ	NHC thermostat	Pseudo-First-Order	Langmuir isotherm	LDH/graphene	(13.1, 13.55, and 14.1) mg/g	This Paper
AC, CAF, and SMZ	NHC thermostat	Pseudo-First-Order	Langmuir isotherm	LDH/g-C_3_N_4_	(11.55, 11.88, and 12.1) mg/g	This Paper
AC	MD simulation	Linear Lumped Resistance	Langmuir isotherm	Sugarcane bagasse (SB) and corn cob (CC)	(0.32 for SB and 0.47 for CC) mg/g	[41]
TC	NVT	Pseudo-second-order	Langmuir isotherm	Na-mont	0.92 mmol/g	[42]
SMZ	NVT	Pseudo-second-order	Freundlich isotherm	Na-mont	0.02 mmol/g	[42]
TC	Experimental	Pseudo-second-order	Langmuir and Temkin isotherm	GO	0.65 mmol/g	[43]
TC	Experimental	Pseudo-second-order	Langmuir	GO-MPs	0.08 mmol/g	[44]
TC	Experimental	Pseudo-second-order	Langmuir	Chitosan	0.04 mmol/g	[45]
SMZ	Experimental and DFT calculations	First- and second-order kinetic models	Langmuir, Freundlich, and Prausnitz-Radke	Activated carbon	1.3 mmol/g	[46]

## Data Availability

All data generated or analyzed during this study are included in this published article.

## Supplementary Materials

The following supporting information can be downloaded at: https://www.mdpi.com/article/10.3390/ijms252312730/s1.
