# Supplementary material for: The Electronic Properties and Adsorption Performance of LDH/Graphene, and LDH/g-C3N4 for the Removal of Pharmaceutical Contaminants: A Molecular Dynamics Simulation
Source: Int J Mol Sci. 2024 Nov 27;25(23):12730. doi: 10.3390/ijms252312730 (PMC11641474; doi:10.3390/ijms252312730)
Supplement: Supplementary file 1 [file ijms-25-12730-s001.zip › ijms-3218517-supplementary.pdf]

Supplementary materials for

The Electronic Properties and Adsorption Performance of LDH/graphene, and LDH/g-C_3N_4 for the Removal of Pharmaceuticals Contaminants: A Molecular Dynamics Simulation

Qusai Ibrahim[1], Salem Gharbia[1*]

[1]Atlantic Technological University, Sligo F91 YW50, Ireland

Email: correspondence-salem.gharbia@atu.ie

Table S1. Lattice constants for g-C_3N_4, graphene, and LDH.

Nanocomposite		G-C_3N_4	graphene	LDH
Lattice constants (Å)	a	4.78	4.26	10.5
	b	4.78	2.46	10.24
	c	7.08	25	12.56
Corresponding angle	α	90.00 °	90.00 °	84.00 °
	β	90.00 °	90.00 °	90.00 °
	γ	120.00 °	90.00 °	90.00 °

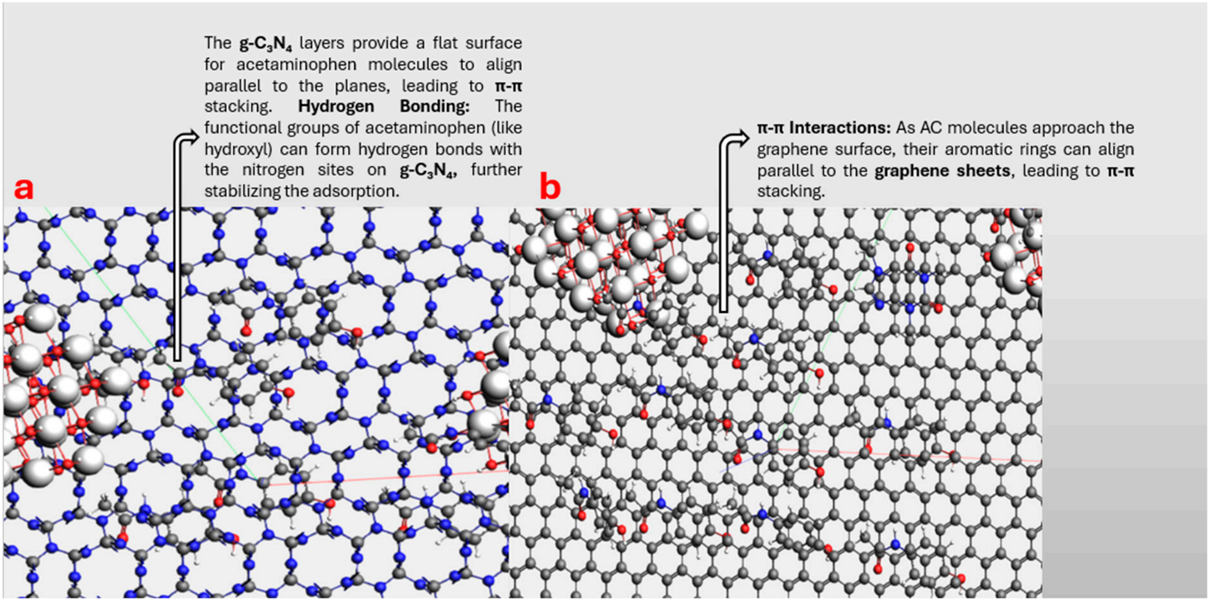

Fig. S1 Illustration of adsorption mechanism of AC molecules on a) LDH/g-C_3N_4, and b) LDH/graphene.

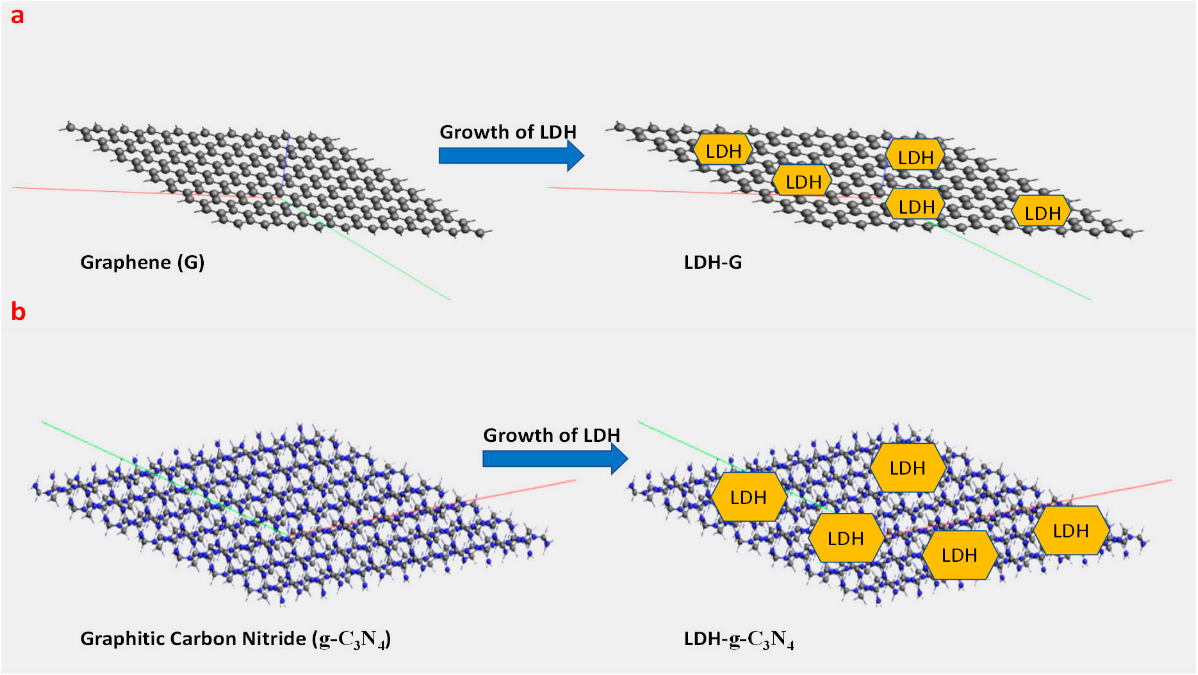

Fig. S2. Initial design of the new nanocomposite materials: a) LDH/graphene, and b) LDH/g-C_3N_4. Carbon atoms in grey, nitrogen atoms in blue, and hydrogen atoms in white.

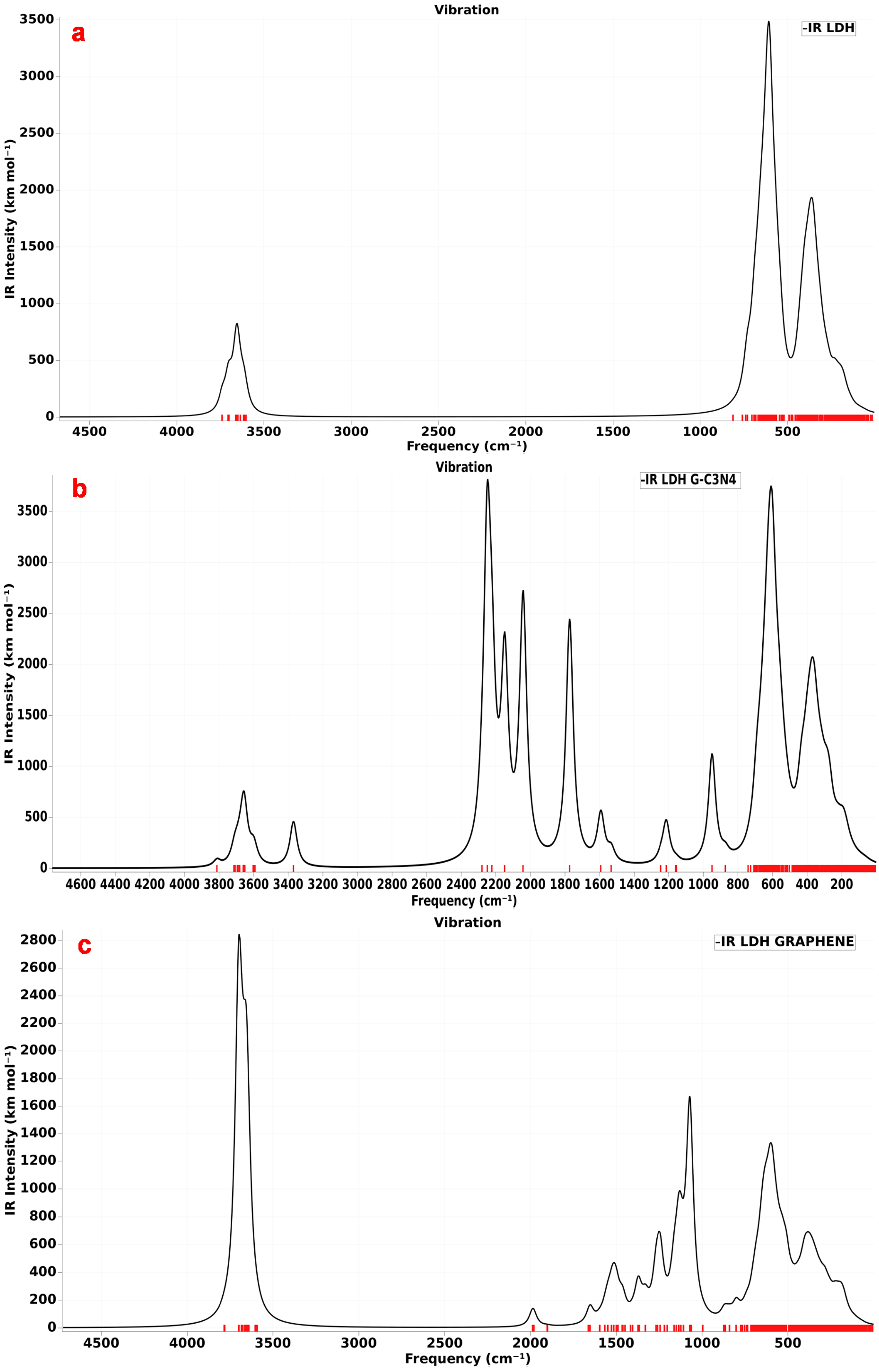
Vibration
a
-IR LDH
IR Intensity (km mol⁻¹)
Frequency (cm⁻¹)
Vibration
b
-IR LDH G-C3N4
IR Intensity (km mol⁻¹)
Frequency (cm⁻¹)
Vibration
c
-IR LDH GRAPHENE
IR Intensity (km mol⁻¹)
Frequency (cm⁻¹)

Fig. S3 The vibrational frequencies, IR intensity calculated by DFTB code for a) LDH, b) LDH/g-C_3N_4, and C) LDH/graphene.

Table S2. Geometry optimization parameters for LDH/g-C_3N_4.

Iteration	Current energy (Hartree)	Energy change (Hartree)	Gradient max.	Gradient rms.
1	-159.667	-	1.664	0.1498
250	-182.0307	-22.3637	0.0413	0.00658
500	-183.3312	-1.3	0.0678	0.0057
750	-185.122	-1.7908	0.1024	0.00967
1000	-185.606	-0.484	0.301	0.01067
1500	-185.7407	-0.1347	0.2964	0.0102
2000	-185.9174	-0.1767	0.3114	0.01020
2500	-186.0231	-0.1057	0.0626	0.00211
3000	-186.1488	-0.1257	0.3063	0.0105
3500	-186.2176	-0.0688	0.0697	0.00261
4000	-186.2826	-0.065	0.30766	0.01137
4500	-186.301	-0.0184	0.071	0.00282

Table S3. Geometry optimization parameters for LDH/graphene.

Iteration	Current energy (Hartree)	Energy change (Hartree)	Gradient max.	Gradient rms.
1	-405.726	-	1.144	0.0637
250	-418.98	-13.254	0.3828	0.0178
500	-419.803	-0.823	0.1365	0.0176
750	-420.842	-1.039	0.1177	0.0159
1000	-421.201	-0.359	0.0989	0.0148
1500	-422.118	-0.917	0.0778	0.0141
2000	-423.019	-0.901	0.0818	0.0137
2500	-423.556	-0.537	0.1012	0.0128
3000	-424.331	-0.775	0.0991	0.00312
3500	-425.012	-0.681	0.213	0.0311
4000	-425.112	-0.1	0.1199	0.0341
4143	-425.191	-0.079	0.2766	0.00453

Table S4. Wave numbers and assignment of bands in the IR spectra of LDH nanocomposite material.

Frequency (cm^{-1})	Stretching	Bending	Assignment
608	✓	-	Na-O
632	-	✓	O-H

665	-	✓	O-H
730	-	✓	O-H
3616	✓	-	O-H
3655	✓	-	O-H
3740	✓	-	O-H

Table S5. Wave numbers and assignment of bands in the IR spectra of LDH/g-C_3N_4 nanocomposite material.

Frequency (cm^{-1})	Stretching	Bending	Assignment
434	✓	-	Na-O
696	-	✓	Na-O-H
950	-	✓	Na-O-H
1215	✓	-	C-N
2250	✓	-	C-H
3370	✓	-	O-H
3602	✓	-	O-H

Table S6. Wave numbers and assignment of bands in the IR spectra of LDH/graphene.

Frequency (cm^{-1})	Stretching	Bending	Assignment
509	✓	-	Na-O
801	-	✓	Na-O-H
1550	✓	-	C-C
1987	✓	-	C-C
3650	✓	-	O-H
3653	✓	-	O-H
3700	✓	-	O-H

Table S7. Atomic charges of AC, CAF, and SMZ on LDH/g-C_3N_4.

Adsorption Molecule	Atom	Adsorption state	Charge (e)
AC	C	Before	-0.192

		After	0.21
	H	Before<br>After	0.208<br>0.221
	O	Before<br>After	-0.505<br>0.12
	N	Before<br>After	-0.53<br>0.33
CAF	C	Before<br>After	-0.230<br>0.23
	H	Before<br>After	0.070<br>0.12
	O	Before<br>After	-0.28<br>0.141
	N	Before<br>After	-0.235<br>0.38
SMZ	C	Before<br>After	-0.29<br>0.23
	H	Before<br>After	0.12<br>0.21
	O	Before<br>After	-0.33<br>0.29
	N	Before<br>After	-0.54<br>0.99
	S	Before<br>After	0.299<br>1.12

Table S8. Atomic charges of AC, CAF, and SMZ on LDH/graphene.

Adsorption Molecule	Atom	Adsorption state	Charge (e)
AC	C	Before<br>After	-0.22<br>0.23
	H	Before<br>After	0.17<br>0.228
	O	Before	-0.589

		After	0.177
	N	Before<br>After	-0.588<br>0.36
CAF	C	Before<br>After	-0.2<br>0.26
	H	Before<br>After	0.11<br>0.19
	O	Before<br>After	-0.29<br>0.24
	N	Before<br>After	-0.239<br>0.48
SMZ	C	Before<br>After	-0.2<br>0.211
	H	Before<br>After	0.17<br>0.33
	O	Before<br>After	-0.35<br>0.31
	N	Before<br>After	-0.59<br>1.1
	S	Before<br>After	0.32<br>1.27

Table S9. Number of adsorbed AC, CAF, and SMZ molecules on LDH/g-C_3N_4.

Time (Ps)	AC	CAF	SMZ
5	40	33	48
10	65	60	73
15	75	80	88
20	86	90	95
25	93	95	102
30	98	100	106

35	105	107	110
40	106	108	114
45	107	109	116
50	108	110	118
55	108	110	120
60	108	111	120
65	109	110	120
70	108	110	120

Table S10. Number of adsorbed AC, CAF, and SMZ molecules on LDH/graphene.

Time (Ps)	AC	CAF	SMZ
5	45	40	55
10	70	68	79
15	85	88	94
20	97	100	111
25	110	115	125
30	117	123	132
35	122	127	136
40	123	128	140
45	124	129	142
50	124	129	142
55	123	128	141
60	124	129	142
65	124	129	142
70	124	129	142
